# Examining How Interaural Differences Owing to Head Rotation during Walking Improve the Distance of Auditory Obstacle Perceptions among Individuals with Visual Impairment: A Case Study in Small-Scale Blind Group

**DOI:** 10.3390/life14030356

**Published:** 2024-03-07

**Authors:** Takahiro Miura, Naoyuki Okochi, Masaki Matsuo, Ken-ichiro Yabu, Atsushi Katagiri, Keiichi Yasu, Masatsugu Sakajiri, Tohru Ifukube

**Affiliations:** 1 National Institute of Advanced Industrial Science and Technology (AIST), Kashiwa 277-0882, Japan; 2 Research Center for Advanced Science and Technology (RCAST), The University of Tokyo, Tokyo 153-8904, Japan; okochi@bfp.rcast.u-tokyo.ac.jp (N.O.); yabu@human.iog.u-tokyo.ac.jp (K.-i.Y.); ifukube@human.iog.u-tokyo.ac.jp (T.I.); 3 Faculty of Health Science, Tsukuba University of Technology, Tsukuba 305-0005, Japan; matsuo@cs.k.tsukuba-tech.ac.jp (M.M.); sakajiri@cs.k.tsukuba-tech.ac.jp (M.S.); 4 Push-Pop Co., Ltd., Kumagaya 360-0816, Japan; pushpop@po.kumagaya.or.jp; 5 Faculty of Industrial Technology, Tsukuba University of Technology, Tsukuba 305-0005, Japan; k-yasu@a.tsukuba-tech.ac.jp

**Keywords:** visually impaired people, obstacle sense, interaural difference, coloration

## Abstract

The ability of individuals with visual impairment to recognize an obstacle by hearing is called “obstacle sense”. This ability is facilitated while they are moving, though the exact reason remains unknown. This study aims to clarify which acoustical factors may contribute to obstacle sense, especially obstacle distance perception. First, we conducted a comparative experiment regarding obstacle distance localization by individuals who are blind (N = 5, five men with blindness aged 22–42 (average: 29.8)) while they were standing and walking. The results indicate that the localized distance was more accurate while walking than while standing. Subsequently, the head rotation angle while walking and acoustic characteristics with respect to obstacle distance and head rotation angle were investigated. The peaks of the absolute head rotation angle during walking ranged from 2.78° to 11.11° (average: 6.55°, S.D.: 2.05°). Regarding acoustic characteristics, acoustic coloration occurred, and spectral interaural differences and interaural intensity differences were observed in the blind participants (N = 4, four men including two blind and two control sighted persons aged 25–38 (average: 30.8)). To determine which acoustic factors contribute, we examined the threshold of changes for interaural differences in time (ITD) and intensity (IID) (N = 11, seven men and four women with blindness aged 21–35 (average: 27.4)), as well as coloration (ICD) (N = 6, seven men and a woman with blindness aged 21–38 (average: 29.9))—depending on the head rotation. Notably, ITD and IID thresholds were 86.2 μs and 1.28 dB; the corresponding head rotation angles were 23.5° and 9.17°, respectively. The angle of the ICD threshold was 6.30° on average. Consequently, IID might be a contributing factor and ICD can be utilized as the cue facilitating the obstacle distance perception while walking.

## 1. Introduction

Various special support education and rehabilitation methods and assistive devices have been proposed to support the daily lives of visually impaired persons [1,2,3,4,5,6,7]. Particularly in gait training, which is carried out for them to acquire independent mobility, the main focus is on orientation and mobility (O&M) [8,9,10,11,12]. Practice and research on O&M abilities have been conducted with congenitally and acquired visually impaired individuals of various ages [13,14,15,16,17,18,19,20,21,22].

On the other hand, most individuals who are blind recognize the existence of objects and even unreachable silent objects using auditory information [8,9,23,24,25,26,27,28]. The ability is termed *obstacle sense* [24,25,28,29,30]. They may recognize these objects by acoustical changes, such as the reflection [30,31,32,33,34,35], absorption, or insulation [34,36,37,38] of various sounds in the real environment, e.g., traffic sound, voices of other people, and other continuous noises [39].

Additionally, body movement may enable them to perceive obstacle distance more accurately as well as source distance perception [35,40], similar to the improvement of sound localization through head movements [41,42,43]. Rosenblum et al. conducted a study on accuracy differences in obstacle distance when blindfolded sighted participants only stood and also when they moved [40]. They found that their distance discrimination is more accurate in the moving case than in the standing case. Miura et al. reported that binaural listening with head rotation helps blind persons enhance their ability to accurately perceive even the existence and distance of narrow obstacles [35]. However, it remains unknown how acoustical cues are caused by body movement and head rotation as well as how they can contribute to accurate obstacle distance perception. If the mechanism is ascertained, the findings would be useful for a systematic rehabilitation method of auditory orientation and for a design of blind mobility aids.

Therefore, this study aims to clarify which acoustical factors can contribute to obstacle sense, especially obstacle distance perception. First, we conducted experiments on the accuracy of obstacle distance perception in two conditions: static condition, where participants were just standing without head movement, and dynamic condition, where they were walking toward a target. From the results, we assumed that higher accuracy in the dynamic condition is caused by the acoustic changes associated with approaching the obstacle, and head rotation resulting in interaural differences. To assess the kind of interaural difference that is used as the cue for obstacle distance perception, we observed angles of head rotation while walking. Third, we conducted a discrimination task for interaural differences in time (ITD), intensity (IID), and acoustic coloration (ICD). Finally, we assumed from the results of the second and third experiments that the interaural difference caused by head rotation while walking should be one of the cues of the obstacle distance perception by individuals who are blind.

## 2. Obstacle Distance Perception in Static and Dynamic Condition

In this section, the localization accuracy of the obstacle distance in blind participants is compared between the static and dynamic condition. Furthermore, we derive an assumption regarding the mechanism of the superiority.

### 2.1. Participants

Three congenitally blind participants and two participants who developed blindness of ages 22–42 years (average: 29.8) participated in the experiment. Three were totally blind, while one could only recognize light stimuli. Although the number of participants in the experiment is not large, some previous studies have included this number of participants [31,33,38,44]. All participants were male and had normal hearing (<20 dB in hearing level), that is, within normal limits in pure-tone audiometry (Rion AA-75). Although they had not undergone speech audiometry or otoacoustic emission testing just before the experiment, they reported no medical history of hearing problems. Before the experiment, the participants who could perceive light stimuli wore eye masks to block visual information.

### 2.2. Experimental Setup

**Figure 1:**
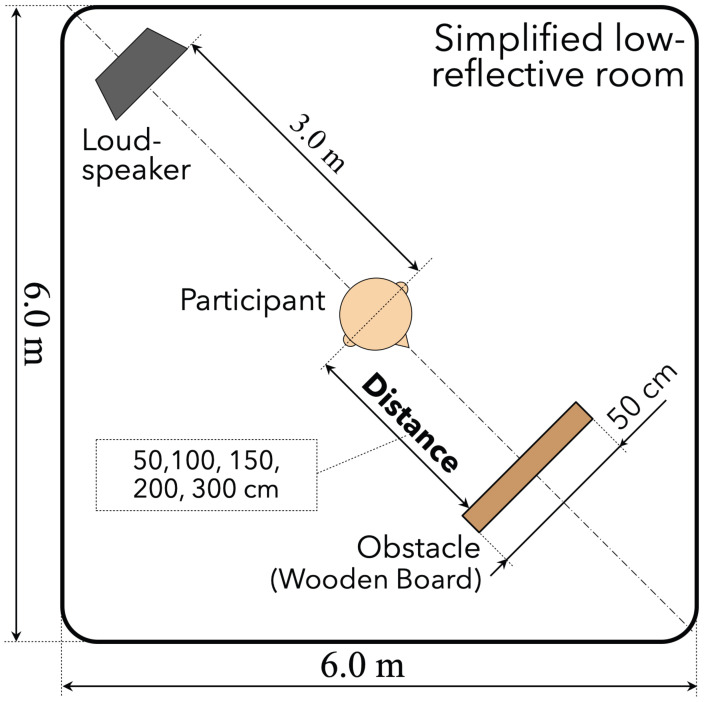
Experimental environment for localization accuracy of obstacle distance.

The experimental environment is illustrated by Figure 1. A loudspeaker (Tannoy, System 800) was set 3.0 m from the center of a silent simplified low-reflective room at the University of Tokyo (size: 6×6×3 m, background noise level: 30 dB, reverberation time: 0.18 s), with the center height of loudspeaker body equal to the center height of each participant’s head. Participants stood at the center of the chamber or started to walk from the center in each trial. This experimental setup is made for the simple model of obstacle distance perception by the cue of the interference between direct and obstacle-reflected sounds, referring to previous studies [25,31,33].

The obstacle was a 70 cm (height) × 50 cm (width) × 2.4 cm (thickness) wooden board and was supported by a wheel-attached tripod with the obstacle’s center height equal to the center height of each participant’s head. The distance of the obstacle from the participants was variable. The positioning of the loudspeaker, the obstacle, and the participant in the experiment was determined based on a similar experiment reported previously [25,28,33,35].

Participants could set the sound intensity of the loudspeaker at a comfortable level (45∼60 dB SPL (sound pressure level)) at the beginning of the experiment. The reason for this setup was to examine their performance under the conditions that they could most easily perceive obstacles, and also to make them feel psychologically safe, thereby providing them with a safety measure during the experiment.

### 2.3. Method

**Figure 2:**
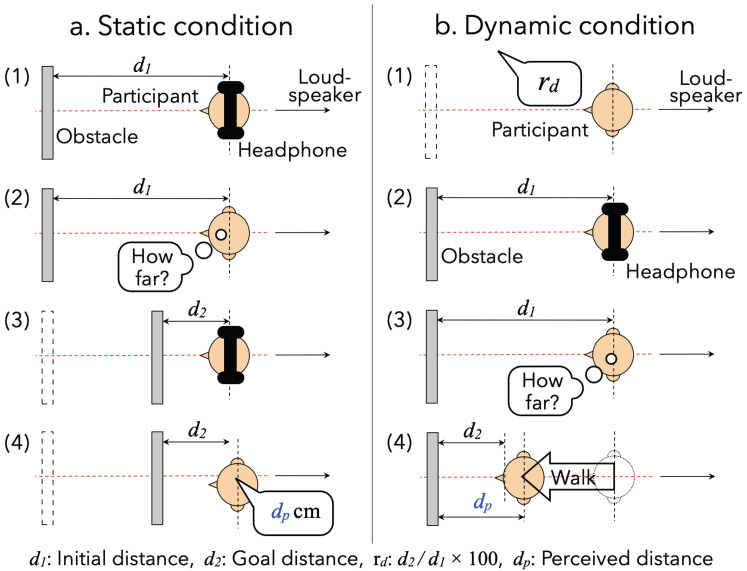
Experimental procedures in static and dynamic conditions.

The experimental procedure is illustrated by Figure 2.

Before starting each trial, the participant was asked to stand at the center of the room as presented in Figure 1 while wearing headphone to shut out environmental sounds. First, the experimenter moved the obstacle to an arbitrary position (initial distance: $d_{1}$): 50, 100, 150, 200, or 300 cm from the participant. Second, pink noise (frequency range: 20–20,000 Hz) was generated from the loudspeaker because ambient noise in the real environment is closer to pink noise than it is to white noise. Also, some studies regarding localizations of sound sources and obstacles used pink noise as a sound source [35,45,46,47,48]. Then, the participant listened to the sound after taking the headphones off. Third, the participant performed the following tasks in either static or dynamic conditions:

**Static:**  the participant was asked to wear headphones again while the experimenter moved the obstacle at the position of various distances (second distance: $d_{2}=d_{1}\times r_{d}/100,∵r_{d}$: percentage ratio between initial and second distances (=25,50, or 75)). Thereafter, the participant listened to the sound and answered the perceived distance $d_{p}$ with cm unit after taking the headphones off (see the left column of Figure 2).
**Dynamic:**  the participant was asked to move to the position of $r_{d}(=25,50$, or 75) from $d_{1}$. The participants were allowed to move toward and away from the obstacle but were asked not to utter any sound or produce the sound of footsteps while moving. Thereafter, the experimenter checked the arrival distance ($d_{p}$). Such a method of giving them a distance ratio and asking them to move to the point where the subjective distances match was based on a previous study [33] (see the right column of Figure 2).

The experiment was composed of 5 ($d_{1}$) × 3 ($r_{d}$) × 2 (repeat count) = 30 times at each conditions. Two participants carried out the static condition first.

Static and dynamic conditions reflect the methods of the magnitude estimation and the magnitude production, respectively. Although it may be unacceptable to simply compare the results in these conditions, we did so because there is no superiority in these results obtained in two conditions in general [49,50].

### 2.4. Analysis

The perceived obstacle distance ($d_{p}$) and the ratio of perceived and goal obstacle distances ($d_{p}/d_{2}$) were first plotted in the aforementioned static and dynamic conditions. Then, an analysis of variance (ANOVA) was employed to identify significant differences among responses to $d_{p}$ and $d_{p}/d_{2}$ and to examine the main effects of conditions, initial distances, and $r_{d}$ for these responses. Before the ANOVA was performed, aligned rank transform (ART) [51,52] was conducted on the scales as the responses were non-normally distributed. Then, the significance of the main effects was determined using post hoc multiple comparison methods based on the least-square means and Tukey’s multiplicity adjustment [53,54]. Then, we calculated the effect sizes, such as partial $\eta^{2}$ and Cohen’s *d*, and determined the degree based on Cohen’s effect size indices [55].

### 2.5. Results

#### 2.5.1. Perceived Distance of an Obstacle

**Figure 3:**
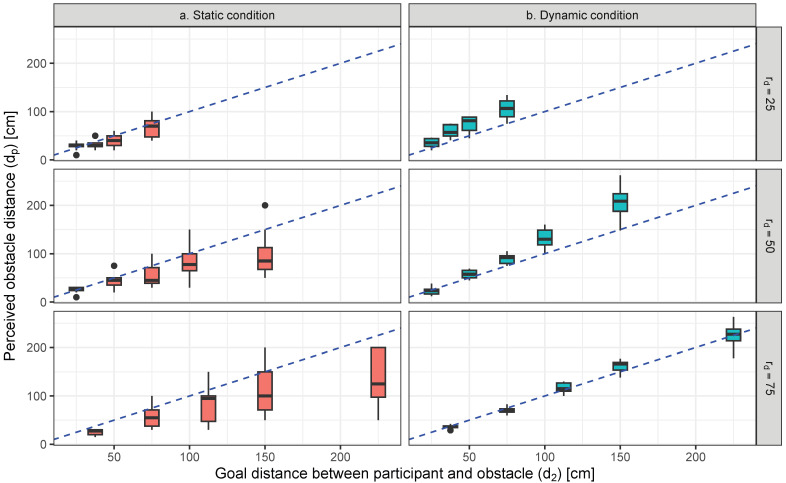
Perceived obstacle distance ($d_{p}$) in static and dynamic conditions at each distance ratio ($r_{d}$) of initial and goal distances ($d_{2}$). The dashed lines indicate the goal distance between the participant and obstacle.

The perceived distance ($d_{p}$) at each distance ratio of initial and goal distances ($r_{d}$) in static and dynamic conditions is illustrated by Figure 3.

An ANOVA performed on changes in $d_{p}$ confirmed a significant main effect for all factors, including static/dynamic conditions, initial distance ($d_{1}$), and $r_{d}$ (p<0.05). The statistical value of these significant factors in $d_{p}$ was as follows: static/dynamic conditions: $F\left(1,1\right)=140.5,\;p<0.001,\mathrm{partial}\eta^{2}=0.41$ (large); $d_{1}$: $F\left(1,4\right)=168.2,\;p<0.001,\;\mathrm{partial}\eta^{2}=0.77$ (large); $r_{d}$: $F\left(1,2\right)=76.2,\;p<0.001,\;\mathrm{partial}\eta^{2}=0.43$ (large). Also, the interactions among all factors were significant or marginally significant: static/dynamic conditions and $d_{1}$: $F\left(1,4\right)=18.3,\;p<0.001,\;\mathrm{partial}\eta^{2}=0.27$ (large); static/dynamic conditions and $r_{d}$: $F\left(1,2\right)=3.24,\;p=0.04<0.05,\;\mathrm{partial}\eta^{2}=0.03$ (small); $d_{1}$ and $r_{d}$: $F\left(1,7\right)=6.26,\;p<0.001,\;\mathrm{partial}\eta^{2}=0.18$ (large); these three factors: $F\left(1,7\right)=1.76,\;p=0.097<0.10,\;\mathrm{partial}\eta^{2}=0.06$ (middle).

The results of multiple comparisons illustrated that the value of $d_{p}$ was significantly larger under dynamic conditions than with static conditions (t(168)=10.367, p<0.001, d=1.50 (large)). The perceived distances in static and dynamic conditions are significantly different (p<0.05, d>1.0 (large)) in all sets of the combination of $d_{1}$ and $r_{d}$, excluding the cases $d_{1}=100$, where $r_{d}=25$ and 75. Also, Figure 3 indicates that perceived distances of static and dynamic conditions are farther and nearer from the goal distance in every $r_{d}$, respectively. As $r_{d}$ increases, $d_{p}$ of the dynamic condition becomes nearer to goal distance, while that of the static condition is more independent of the change in $r_{d}$.

#### 2.5.2. The Ratio of the Perceived and Goal Distances of an Obstacle

**Figure 4:**
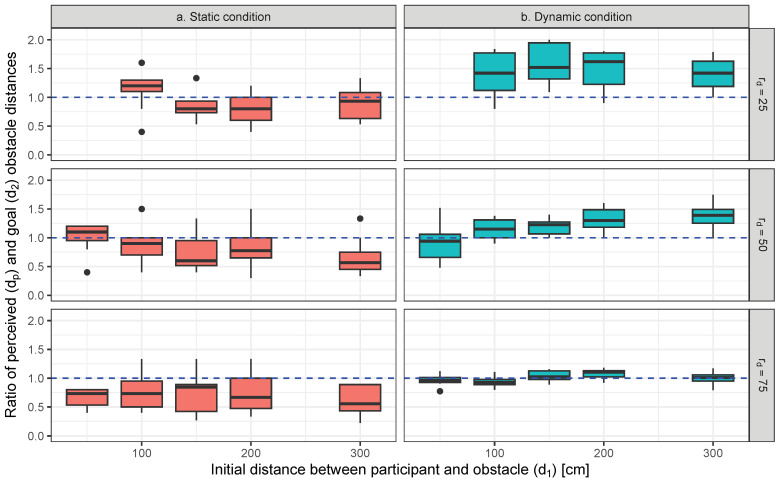
Ratio of perceived and goal obstacle distances in static and dynamic conditions at each distance ratio of initial and goal distances. Marks for the static and dynamic conditions are plotted at ±0.02 m points from the goal distance transverse to each other to facilitate visualizations. The lines with dots indicate the goal distance between the participant and obstacle.

The ratio of the perceived and goal distances ($d_{p}/d_{2}$) is illustrated by Figure 4.

An ANOVA performed on changes in $d_{p}/d_{2}$ confirmed a significant main effect for the static/dynamic conditions, and $r_{d}$ (p<0.05). The statistical value of these significant factors in $d_{p}/d_{2}$ was as follows: static/dynamic conditions: $F\left(1,1\right)=110.2,\;p<0.001,\;\mathrm{partial}\eta^{2}=0.36$ (large); $r_{d}$: $F\left(1,2\right)=22.5,\;p<0.001,\;\mathrm{partial}\eta^{2}=0.19$ (large). The interaction between static/dynamic conditions and $d_{1}$ was significant for $d_{p}/d_{2}$ ($F\left(1,4\right)=4.87,\;p<0.001,\;\mathrm{partial}\eta^{2}=0.09$ (middle)).

The results of multiple comparisons illustrated that the value of $d_{p}/d_{2}$ was significantly larger under dynamic conditions than with static conditions (t(168)=10.564, p<0.001, d=1.52 (large)). The ratio of perceived and goal obstacle distances in static and dynamic conditions was also significantly different (p<0.05, d>1.0 (large)) in all sets of the combination of $d_{1}$ and $r_{d}$, excluding the cases $d_{1}=100$, where $r_{d}=25$ and 75, which is the same result as the perceived distance. In the static condition, the median of $d_{p}/d_{2}$ at $r_{d}=25$ is nearer to 1.0 than that at $r_{d}=50$ or 75; there is no significant difference between $r_{d}=50$ and 75. On the contrary, in the dynamic condition, as $r_{d}$ increases, the median of $d_{p}/d_{2}$ tends to near 1.0; there are significant differences among $r_{d}=25$, 50, and 75 (p<0.001, d>1.0 (large)). Also, with the increase in $r_{d}$, the interquartile range of $d_{p}/d_{2}$ in the dynamic condition becomes lower than that in the static condition.

### 2.6. Discussion

As presented in Figure 4, with a decreasing $r_{d}$, $d_{p}/d_{2}$ in the dynamic condition is farther from the goal distance, and its interquartile range becomes larger. However, the variability of the interquartile range of perceived distances presented in Figure 3 in the static condition is larger than that in the dynamic condition. Additionally, the differences between the standard deviation $d_{p}/d_{2}$ of the two conditions becomes smaller as $r_{d}$ decreases, as shown in Figure 4. Thus, the differences in the distance localization of the obstacle between the two conditions may become small when there is a large change in obstacle distance.

When $r_{d}$ is higher (such as $r_{d}=75$), $d_{p}/d_{2}$ is nearer to 1.0, and its interquartile ranges become smaller in the dynamic condition. Rosenblum obtained that blindfolded participants can perceive the obstacle distance more correctly during the moving condition compared to the static condition [40]. The dynamic condition may be superior in obstacle distance localization in the case of a moving small distance (such as the condition of $r_{d}=75$). The participants also reported that the dynamic condition was better when determining the obstacle distance because it allowed for moving heads back and forth and rotating in order to listen to the acoustic circumstance during walking. The participants might utilize the acoustic changes by walking, and the vestibular stimulation may be produced by the self-motion. Pettorossi et al. assumed that quick localization with head rotation is caused by vestibular stimulation [56]. Obstacle distance perception may also be improved by self-motion.

The participants also stated that some kind of acoustic change can be perceived by head rotation during walking. Further, some acoustic changes may occur in the intensity of reflected sounds from the obstacle and the acoustic coloration (hereafter called ‘coloration’) caused by the interference between direct and reflected sounds [32,33]. Moreover, the head rotation while walking generates various kinds of interaural differences in, e.g., time, intensity, coloration, and other sorts of acoustic changes. Continuous perception of these differences, therefore, may help individuals who are blind to localize obstacles accurately. Interaural difference is related to the accurate directional localization of the sound source and the non-sounding obstacle [35,57]. This difference could also be related to our result of accurate distance localization in the dynamic condition.

However, the extent of the head rotation while walking and the interaural difference in head rotation are specifically unknown. Thus, in the following section, head rotation and interaural differences are measured, with the assumption that head rotation can contribute to more accurate distance obstacle localization.

## 3. Acoustic Change While Walking

### 3.1. Head Rotation While Walking

#### 3.1.1. Participants

Two individuals who are blind (a congenitally blind male aged 35 and an acquired blind male aged 38) and two control sighted persons (males aged 25) participated in the experiment. The number of participants who consented to the experiment from the previous section was reduced due to the restrictions on the clothing and the installation of the reflective markers. None had problems walking and none had a history of cervical disorders. We did not measure saccadic conditions such as vestibulo-ocular reflex (VOR) with the video head impact test (vHIT) [58].

#### 3.1.2. Method

**Figure 5:**
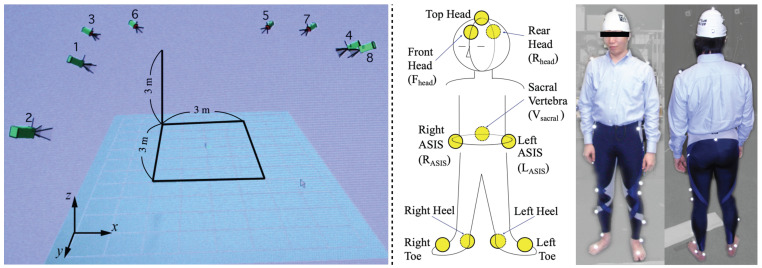
(**Left**) Experimental environment for measuring body movement. Participants were asked to walk in the square area presented. (**Right**) Positions of attached markers for the motion capture measurement of head rotation while walking.

Body movement was measured by a motion capture system (MAC3D, Motion Analysis Corporation). Eight cameras were attached in the experimental room at the University of Tokyo with a measurable range of 3 m × 3 m × 3 m, as presented in the left panel of Figure 5. First, three markers were attached to each participant’s head and two markers to their heels and toes, as illustrated in the right panel of Figure 5. Second, participants were asked to walk in a straight line in a zone where at least three cameras could capture the attached markers. At that time, participants were asked to walk with the awareness that an obstacle was located forward, though there was no obstacle in front of them.

The motion capture system simultaneously recorded all markers’ trajectories [59]. The head rotation was transformed to the angle formed by two markers on a line crossing the front and rear of the head, and the reference axis. In the case of a deficit of markers at the front or rear of the head, a marker on top of the head was used. When coordinate values of the marker positions are defined as presented in the right panel of Figure 5, the temporal head angle θ(t) is calculated as follows:

(1) $$\begin{array}{cc} \theta (t) & =\int_{0}^{t}\tan^{-1}\frac{F_{head,y}\left(t\right)-R_{head,y}\left(t\right)}{F_{head,x}\left(t\right)-R_{head,x}\left(t\right)}dt \end{array}$$

where subscripts such as *x* and *y* are the coordinate values of *x* and *y*. The positive value of θ(t) means counterclockwise rotation from the top view.

Additionally, the movement of both legs while walking was measured by the altitude of heel-attached markers.

#### 3.1.3. Results

**Figure 6:**
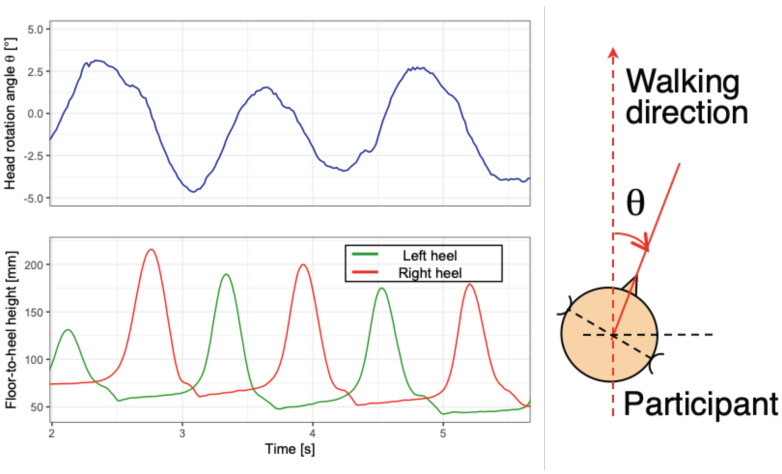
An example of head rotation while walking by a blind participant. Upper left graph represents the head rotation angle θ(t) in relation to time. The positive value of θ(t) means counterclockwise rotation from the top view, as shown in the right diagram. Lower left graph represents footsteps at the same time; the vertical coordinate is the heel height from the floor.

An example of head rotation while walking is presented in Figure 6. The results for both participants who are blind and sighted were not significant (t(16.39)=−0.75, p=0.465>0.10, Welch test). The direction of head rotation is opposite to the leg direction in the swing phase. It was ascertained that the head rotation increased, and the peak-to-peak head rotating angle varied in the range of 2.78°–11.11° (average: 6.55°, S.D.: 2.05°, median: 6.78°).

### 3.2. Acoustic Changes in Relation to the Obstacle Distance and Head Rotation Angle

#### 3.2.1. Method

Acoustic measurement was performed in the experimental environment as presented in Figure 1. First, the dummy head mounting microphones at both ears (B&K 4128 HATS) was placed at the center (the location of “participant”) of the silent simplified low-reflective room. Second, the obstacle was located at the distance of 12.5, 25, 37.5, 50, 75, 100, 150, and 200 cm from the center of the interaural axis of the dummy head, with the obstacle’s center height equal to the center height of the head part of the dummy head. At that time, the angles of the line connecting the ears of the dummy head to the horizontal surface of the obstacle were 3, 6, and 11 degrees, based on the head rotation angles obtained in the previous section.

Thereafter, the measurement signal was fed to the loudspeaker from a computer via an audio unit (FA-66) and amplifier (Time Domain YA1). As the signal, swept-sine (known as time-stretched pulse, TSP) [60,61,62] with a duration of 1.49 s (65,536 points) was employed. The signal from the loudspeaker was detected by the microphones at HATS and was recorded into the memory of the computer via the audio unit. The sampling frequency and quantization bit rate in both the input and output of the laptop were 44.1 kHz and 16 bit, respectively.

The measurement items were as follows: spectral difference between acoustic transfer functions with and without obstacle in relation to the obstacle distance and head rotation angle, interaural spectral difference between spectral differences in left and right ear when an obstacle existed, and interaural intensity difference (IID) (also known as interaural level difference (ILD)). IID is calculated by the maximum intensity value of impulse responses.

#### 3.2.2. Result

**Figure 7:**
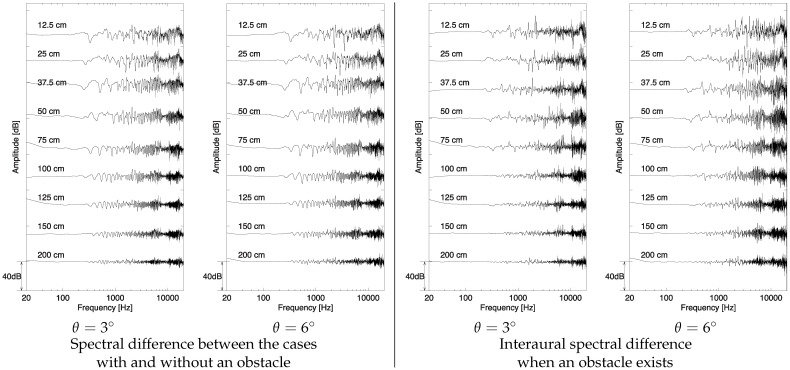
Examples of (**Left**) spectral difference and (**Right**) interaural spectral difference. Labels near each lines suggest obstacle distance.

Examples of spectral differences and interaural spectral differences in relation to obstacle distance are presented in Figure 7 (head rotation angle θ was set to 3° or 6°). The spectral differences in the upper figures of Figure 7 were obtained by the subtraction of acoustic transfer functions between with and without the obstacle measured by the dummy head (left ear). These lines were smoothed by a triangular filter (50 points). Figure 7 presents the interaural spectral differences obtained through the subtraction between the spectral differences measured at left and right ears.

As presented in the left figure of Figure 7, spectral differences have successive comb-and-dip characteristics; this effect is also reported by Seki et al. [32,33]. The absolute values of spectral differences decreased with the increase in obstacle distance. When the head rotation angle is 3°, the average peak-to-peak amplitude differences at combs and dips under 1000 Hz at 12.5, 25, 37.5, 50, 75, 10, 125, 150, and 200 cm of obstacle distance are approximately 20.4, 20.8, 20.4, 14, 12.4, 11.6, 8.8, 7.6, and 4.0 dB, respectively.

Additionally, interaural spectral differences illustrated by the below Figure 7 are larger as the head rotation angle increases. The absolute values of interaural differences become decreased with an increase in obstacle distance. When the head rotation angle is 3°, the average peak-to-peak at combs and dips under 1000 Hz at 12.5, 25, 37.5, 50, 75, 10, 125, 150, and 200 cm of obstacle distance is 15.6, 14.8, 12.4, 8.4, 7.2, 3.6, 2.4, 2, and 1.6 dB, respectively.

**Figure 8:**
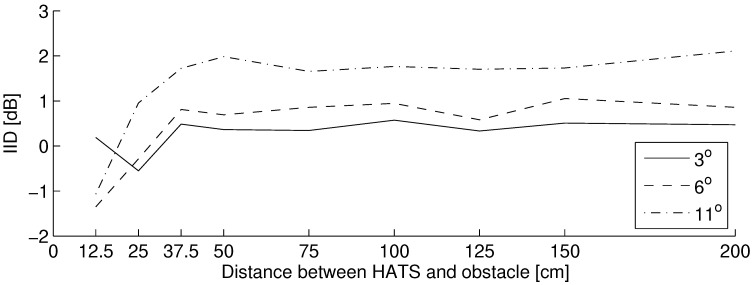
Interaural intensity differences in relation to obstacle distance at each head rotation angle.

**Figure 9:**
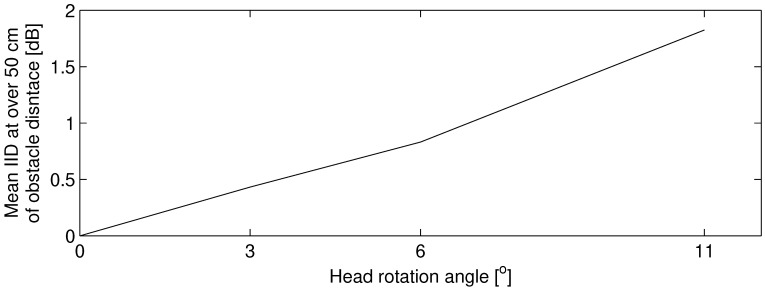
Mean interaural intensity differences at over 50 cm of obstacle distance in relation to head rotation angle.

Interaural intensity differences in relation to obstacle distance are presented in Figure 8. In this graph, lines are plotted for every head rotation angle. The IID varied by −0.55∼0.57, −1.35∼1.05 and −1.07∼2.11 dB in 3°, 6° and 11° of the head rotation angle, respectively. At over 50 cm in obstacle distance, IIDs are almost constant with obstacle distance. The mean IID at over 50 cm of distance presented in Figure 9 monotonically increases with an increase in the observed range of head rotation angle. The absolute value of IID is higher when the head rotation angle is larger. However, the distance where positive and negative values of IID are reversed varies in head rotation angle: almost 25 cm in 3° while almost 12.5 cm in 6° and 11°.

### 3.3. Discussion

As presented in the result of spectral differences presented in the left graphs in Figure 7, combs and dips whose intervals changed as the obstacle distance changed are observed. Seki et al. reported on the possibility of the contribution of acoustic coloration caused by the interference between the direct and reflected sounds to obstacle distance perception by individuals who are blind [31,32]. Additionally, Kates et al. found that a noticeable spectral ripple caused by the interference between the sound and the sound with delay varied by 2.15–3.05 dB in peak-to-peak when the delay varied by 1.8–17.5 ms [63], which responds to 30–300 cm in obstacle distance. Consequently, participants could determine the obstacle distance by the cue of this acoustic change. The interaural difference in spectral differences illustrated by the below figures of Figure 7 increases with the increase in head rotation angle. These interaural differences can be one of the criteria for the high accuracy of obstacle distance localization. This assumption is discussed in the subsequent section.

As presented in Figure 8, the reverse change of the positive and negative value in IID is over 1.0 dB in measured head rotation angle. Hartmann reported that the just noticeable level (JND) of IID in white noise is, at largest, 1 dB [64]. Consequently, when the JND of IID in individuals who are blind is the same as that in those who are sighted, as reported, there is no significant difference in the peripheral nervous system between them [33], while head rotation can be perceived and used to localize obstacle distances less than approximately 25∼50 cm: this range includes the “final appraisal", which is reported by Supa et al. [25,30]. The difference limen (DL) of IID is investigated in the subsequent section.

Additionally, the positive and negative values of IID are reversed depending on the combination of obstacle distance and head rotation angle. The intensity relationship between the direct and reflected sounds is changed as the head rotation angle changes. Thereafter, the intensity of reflected sound may be larger than that of direct sound at particular obstacle distances, which is decided by the head rotation angle. The IID in 3° of head rotation angle at 12.5 cm of distance is almost the same as IIDs at 50 cm. This may be because the intensity of reflected sounds at both ears shows little difference.

Walking with head rotation, as shown in Figure 6, results in intermittent acoustic changes with or without interaural differences. Such acoustic changes could be used for obstacle localization in combination with ambiguous visual information in people with severe low vision. Notably, when such people approach a thin object such as a utility pole, intermittent perception of visual and auditory symmetries [65,66] and asymmetries may improve efficiency in continuing to localize the object. Such experience and training prior to acquired total blindness may improve the ability to perceive obstacles with hearing in combination with visual experience. However, to ascertain the feasibility of this discussion, it is necessary to confirm whether the interaural differences during walking are perceivable.

## 4. Discrimination Experiment for Interaural Differences

To assess which interaural differences can be used as cues of obstacle distance perception during walking, discrimination thresholds of interaural differences were investigated in blind participants. Surveyed interaural differences involved interaural differences in time (ITD), intensity (IID), and dip-frequency interval in coloration (ICD). ITD was checked to see whether only reflected sound can contribute to the obstacle distance perception.

### 4.1. ITD and IID

#### 4.1.1. Participants

Eight congenitally blind and three acquired blind participants aged 21–35 years (average: 27.4) participated in the experiment. Seven were males and four were females. All participants had normal hearing, within normal limits in pure-tone audiometry. Moreover, although they had not undergone speech audiometry or otoacoustic emissions testing just before the experiment, they reported no medical history of hearing problems.

#### 4.1.2. Sound Stimuli

**Figure 10:**
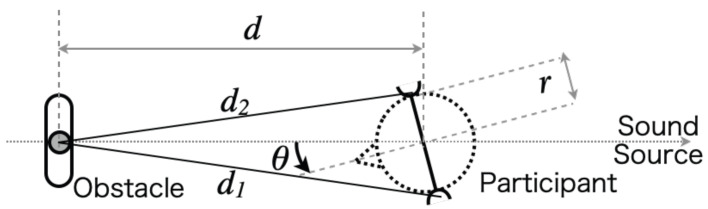
Simulation condition of sound stimuli with interaural differences in time, intensity, and coloration.

ITD varied as follows: 0 μs, 7.5 μs, 15.0 μs, 37.6 μs, 74.8 μs, 147.5 μs, 215.7 μs, and 305.3 μs, which corresponds to 0°, 1°, 2°, 5°, 10°, 20°, 30°, and 45°, respectively, in head rotation angle θ calculated by the following equations.

(2) $$\begin{array}{cc} \Delta t & =\left|\frac{d_{2}\left(\theta \right)-d_{1}\left(\theta \right)}{c}\right|\\ ∵\;d_{k}\left(\theta \right)= & \sqrt{d^{2}+r^{2}+{\left(-1\right)}^{k}2dr\sin\theta }\;\left(k=1,2\right) \end{array}$$

where Δt, *d*, and *r* represent ITD, the distance between the obstacle and head center (set as 1.0 m), and head radius (set as 7.43 cm; this value is calculated by half of the bitragion breadth presented in [67]), respectively, as presented in Figure 10.

IID varied as follows: 1.0, 0.95, 0.90, 0.85, and 0.80 in ratio, which corresponds to 0, −0.44, −0.92, −1.42, and −1.94 in dB. These sounds were generated from pink noise, as employed in the previous sections.

#### 4.1.3. Method

The experiment was performed in a silent soundproof chamber (Rion, AT-81) at the University of Tokyo. Before the experiment started, the experimenter asked the participants to wear headphones (Sony, MDR-Z600) on their ears and to adjust the sound to a comfortable level (45∼60 dB SPL). First, two successive sounds were presented at the time sequence. The sound duration of both ITD and IID was 1.5 s. The first sound did not include the interaural difference, and the second sound included either IID or ITD.

Participants were asked to answer whether or not the two sounds were the same by pressing 1 or 2 on a keyboard. Additionally, the participants were asked to inform the experimenters when they mistyped so that typing errors could be corrected by the experimenter.

The training session comprised 5 trials in both ITD and IID conditions, while actual tests of each condition were composed of 96 and 100 trials, respectively. In ITD or IID conditions, the aforementioned eight or five parameters recorded 2 times (left or right ear lateralized) were repeated 8 or 10 times, respectively, and each presentation order was randomized. All participants firstly responded to the training session and actual test of ITD, and then those of IID.

The training session included three stimuli that had no ITD and no IID in two successive sounds. In the training session, participants practiced only the way to respond to stimuli and were not informed about the correct answers.

The results of participants who responded “not same” over 75% of the time compared to declaring no difference between the first and second sounds were excluded from the summary: one participant was excluded.

The logistic regression model was employed to calculate the adjusted odds ratio (OR) with a 95% confidence interval (CI) for the participants’ responses to the ITD and IID presentation associated with two factors: the head rotation angle corresponding to ITD and IID, and the lateralization condition corresponding to the left or right ears. Then, the logistic regression curve was plotted and the difference limen (DL) was determined at the 75% point of correct rates [68].

#### 4.1.4. Results

**Figure 11:**
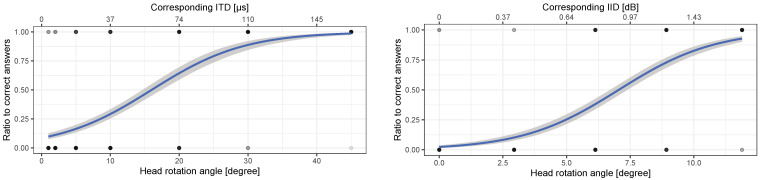
(**Left**) ITD and (**Right**) IID discrimination result as a function of head rotation angles corresponding to ITD and IID, respectively, by participants who are blind. The density of the dots indicates the frequency of responses, and logistic curves were drawn based on these responses.

The left and right figures in Figure 11 show the results of ITD and IID discrimination results, respectively. A logistic regression analysis performed on the responses to ITD and IID changes confirmed a significant main effect for the head rotation angle corresponding to ITD (OR: 1.17 (95%CI: 1.14–1.20), *p* < 0.001) and IID (OR: 1.68 (95%CI: 1.54–1.83), *p* < 0.001), respectively. Also, there were no significant main effects of the responses to ITD and IID changes for the lateralization conditions (*p* > 0.75 in either cases) and no significant interaction between the head rotation angle corresponding to ITD/IID and lateralization conditions (p>0.77 in either cases).

The ITD discrimination result indicated that ITD DL is 86.2 μs, which corresponds to 23.5° in head rotating angle.

On the other hand, the IID DL was 0.847 (1.28 dB), which corresponds to 9.17° in head rotating angle.

#### 4.1.5. Discussion

Regarding the ITD threshold of noise, Klumpp et al. reported that the ITD DL of broadband noise ranged from 5 to 18 μs (average: 10 μs) [69]. They also reported that the mean ITD DL of bandpass noises varied from 9 to 62 μs, e.g., 9 μs of 150–1700 Hz, 14 μs of 425–600 Hz, 44 μs of 2400–3400 Hz, 62 μs of 3056–3344 Hz. Henning reported that ITD DL in bandpass noise tends to change bandwidth-dependently—approximately 20–40, 5–10, 20–50, and 60–70 μs in 100, 600, 1200, and 2500 Hz of bandwidth—when each band was centered on 3900 Hz [70]. Our result, 86.2 μs, was larger than these reported values, which may be due to the fact that the stimulus of the experiment was broadband noise, whose frequency range was 20–20,000 Hz.

Various studies reported that the IID discrimination threshold in noise is, at most, 2.0 dB [64,71,72]. Our result, i.e., 1.28 dB, was consistent with the results of these studies.

From these results, IID may be easier than ITD to perceive from the perspective of head rotation angle and could be used as the cue to perceive the existence and distance of an obstacle when head rotation occurs while accompanying the blind person walking toward an obstacle.

### 4.2. ICD

#### 4.2.1. Participants

Six congenitally blind and two acquired blind participants aged 21–38 years (average: 29.9) participated in the experiment. Seven were males and one was female. All participants had normal hearing, within normal limits in pure-tone audiometry. Moreover, although they had not undergone speech audiometry or otoacoustic emissions testing just before the experiment, they reported no medical history of hearing problems.

#### 4.2.2. Sound Stimuli

A timbre by coloration is caused by the interference between two surrounding sounds around a participant [32,33,73]. One is a surrounding sound directly transmitted to the participant’s ears, and the other is a reflected sound from obstacles in the same surroundings. A time delay between the two sound waves at the participant’s ears forms a comb-like spectral pattern with dips as presented in Figure 7. The frequency difference between the dips is a reciprocal of the time delay. Therefore, the coloration is an important information cue for identifying the distance from the obstacle to the participant.

Here, the dip-to-dip interval in the spectrum of coloration is defined as the dip-frequency interval $f_{d}$, where the relation between $f_{d}$ and the distance from obstacle *d* is as follows:

(3) $$f_{d}=\frac{c}{2d}$$

where *c* stands for sound velocity [33].

Sound stimuli were synthesized by the direct sound from the sound source and reflected sounds from the obstacle. When the distance between both ears and the obstacle, defined as (d+cΔt) or (d−cΔt), in the head rotation angle occurs, ICD is formulated by the following equation in the case where direct sound is broadband noise.

(4) $$\mathrm{ICD}=\frac{2f_{d}^{2}\Delta t}{1-{\left(f_{d}\Delta t\right)}^{2}}$$

Consequently, the relation between the head rotation angle θ and ICD is calculated by (2) and (4). In the experiments described below, we focused on the discrimination threshold of changes in $f_{d}$ for identifying the corresponding thresholds of sound changes in head rotation angle.

#### 4.2.3. Method

The experiment was conducted in a silent soundproof chamber (Rion, AT-81) at the University of Tokyo. Before the experiment, the participants were asked to wear headphones (STAX, SRM-1/MK2 and SR-Λ) on their ears and adjust the sound to a comfortable level (45∼60 dB SPL). This experiment is based on PEST (parameter estimation by sequential testing) [74,75].

**Figure 12:**
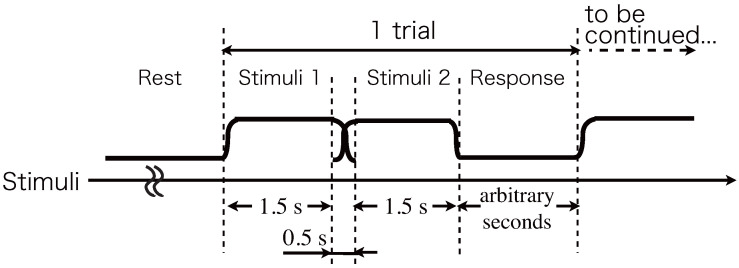
Experimental protocol for discrimination of sound change.

First, two successive sounds were presented at the time sequence presented in Figure 12. The sound duration of two sounds and transition between two sounds were 1.5 s and 0.5 s, respectively. As a stimulus, pink noise was used. The successive two sounds were cross-faded in the transition interval because continuous sound can be perceived when individuals who are blind localize obstacles auditorily while walking.

The first sound was the reference sound and it included no interaural difference. The first sounds were generated in the simulated conditions of 4.0, 3.0, 2.0, 1.5, 1.0, 0.75, 0.5, and 0.3 m of participant-to-obstacle distance, which correspond to 23.3, 17.5, 11.7, 8.8, 5.8, 4.4, 2.9, and 1.8 ms of delay between direct and reflected sound. In these distance conditions, the corresponding intensity of reflected sounds against direct sound was determined as −5.35, −6.45, −7.60, −8.20, −8.65, −8.70, −8.95, and −9.10 dB, respectively, which were calculated by twice the intensity ratio of the coloration threshold of reflected sound reported by Kates [63]. In these cases, the corresponding peak-to-peak differences at combs and peaks are 10.50, 8.99, 7.71, 7.13, 6.74, 6.70, 6.48, and 6.36 dB, respectively. Although the intensities of reflected sounds generally become lower as the participant-to-obstacle distance increases in the real environment, we investigated only ICD thresholds. Thus, we set an equal intensity of reflected sound from the perceptual threshold. The second sound included interaural coloration change. The quantity of ICD in a trial was adaptively determined by the response of the precedent trial, starting with a randomly selected initial ICD.

The stimulus was synthesized by direct and reflected sounds, which have interaural time differences. The time difference between the right and left ear of direct and reflected sounds is 2Δt and −2Δt, respectively. This sound generation technique simulated the amplitude and phase characteristics of coloration.

The participants were asked to answer whether or not the two sounds were the same by inputting 1 or 2 on the keyboard. The experiment consisted of 10 trials for training and arbitrary times for actual tests because of the PEST scheme. In the training session, participants practiced only the way to respond to stimuli, and correct answers were not informed.

#### 4.2.4. Results and Discussion

**Figure 13:**
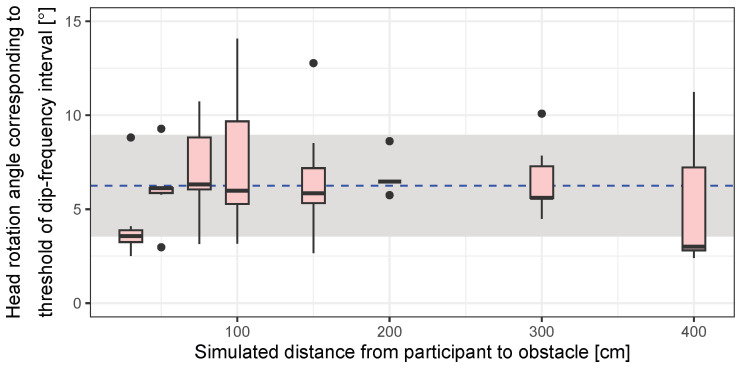
Thresholds of head rotation angle obtained by the threshold of ICD. Blue dashed line and translucent area represent the mean and standard deviation of the thresholds, respectively.

The ead rotation angle calculated by the threshold of ICD is illustrated by Figure 13. The head rotation angle corresponding to ICDs is calculated by (2) and (4). An ANOVA performed on the threshold of ICD confirmed no significant main effect for the simulated participant-to-obstacle distance (F(1,7) = 1.66, *p* = 0.14 > 0.10). Consequently, since the threshold of ICD could be considered as independent from the simulated distance, the thresholds of ICD acquired for all distance conditions were averaged, yielding a mean of 6.30° and an SD of 2.70° (median: 5.97° and interquartile range: 3.52°) as the perceivable angle of ICD.

Compared to the aforementioned results regarding ITD and IID, head rotation angles that could discriminate ICD resulted in the smallest results. Therefore, in a situation where a visually impaired person walks toward an obstacle with some head rotation, the interaural difference in coloration should be one of the most useful clues for the presence and distance of an obstacle, followed by IID and ITD.

## 5. General Discussion

A peak-to-peak head rotating angle occurs at 6.55° on average while walking, as presented in Figure 6, and the head rotation angle corresponding to the threshold of sound change in ITD, IID, and ICD is 23.5°, 9.17°, and 6.30°, respectively, as presented in Figure 11 and Figure 13. Although these are different angles between peak-to-peak and frontal angles, the former situation is generally considered to be easier to perceive because the changes in interaural differences are larger [57]. Thus, individuals who are blind can practically perceive the ICD caused by head rotation while walking, and it is possible to use this interaural difference as a cue for obstacle distance perception.

The IID corresponding to head rotation angle depends on the distance of the obstacle, as presented in Figure 8. When a blind participant is walking at a distance of over 50 cm from the obstacle, the perceivable head rotation angle of IID difference against 0° of head rotation angle (frontal direction to an obstacle) is approximately 9.6° as compared by Figure 9 and Section 4.1.4. This angle is out of range from the average head rotation angle plus–minus standard deviation (4.50°–8.60°), but in the range of the measured head rotation angle (2.78°–11.11°) mentioned in Section 3.1.3. Therefore, individuals who are blind might use the IID as a cue for obstacle distance perception when they occasionally rotate their head more.

The experiment in Section 2 aimed at comparing the accuracy of obstacle distance perception in static and moving conditions, while the experiment in Section 4 aimed at extracting which acoustic factors can be used as cues for obstacle distance perception. The task of Section 2 included more cues for obstacle distance perception, such as self-motion and accompanying vestibular sense. It is true that, as Lewald et al. reported, even passive body rotation significantly influences sound lateralization [76]. They also stated that neck-proprioceptive vibration affects left–right perception: when vibration is presented at the neck on one side, lateralization shifts to the same side [77]. However, Pettorossi et al. indicated that the participant could localize sound sources more quickly when the head was actively rotated rather than passively rotated [56]. These results suggested that self-active motion may sensitize the localization of obstacle position more than passive head rotation associated with walking.

Moreover, although this study focused only on acoustic changes caused by head rotation during walking, in a real environment, the perception of the presence and distance of an obstacle is also based on the perception of the coloration caused by the change in the distance due to the approach to the obstacle. In addition to these acoustic changes caused by approaching and passive head movements, the active nature of these movements can contribute to improving the accuracy of obstacle presence and distance perception. Since this paper did not examine the relationship between these factors and the degree of importance of these factors, this is a subject for future research.

## 6. Limitations

In the experiments in this paper, the loudspeaker was placed only behind the blind participant. However, in a real environment, sound sources are located at various angles and distances, and have various directivity and frequency characteristics. The visually impaired need to selectively listen to usable sounds from stationary or moving sound sources in such conditions. These sounds may occasionally be difficult to distinguish from ambient background noise due to the attenuation. Feierabend et al. reported that the ability to localize frontal sound sources in a noisy environment was significantly lower in blind participants than in sighted participants [78]. Note that the experimental design employed in this study does not include such a process of selective listening, and that participants certainly heard the reflected sound from the obstacle. Since the experiment by Feierabend et al. [78] was a sound source localization task, the localization performance by blind persons may differ in terms of selective listening to localize the distance and direction of obstacles. Moreover, the changes in auditory information associated with head movements, as described in this study, may also contribute to selective listening for sounds related to obstacles. In addition, sound sources located on the listeners’ sides are generally more perceivable than those located in their front or back [79]. It has also been reported that obstacle perception can be achieved even when the sound source is occluded by an obstacle, in addition to when the sound is reflected by the obstacle [34]. Therefore, obstacle sense may still be successfully achieved even when the sound source position is somewhere other than the back of the head, and may even improve with head movement, as shown in the present results. To clarify this matter, further studies are needed to examine the performance of selective listening for obstacle perception and the localization performance of obstacles afterwards in real environments where there are sound sources with various characteristics.

Regarding the characteristics of sound sources, in particular, while the present study examined only a single point-source emitted broadband noise, it should be noted that there are various shapes of sound sources (point, line, and plane sources) with various frequency components in a real environment. Visually impaired people in Japan answered that they have used guide sounds for accessible environments in addition to traffic noise, store noise, and the voices of surrounding people, and they also reported that they can auditorily localize walls, telephones, and traffic poles [80]. Regarding frequency components of a sound source, wideband sounds generally provide the easiest cue for perceiving obstacles in principle [81]. Some researchers reported that the overtone components caused by coloration are easier to perceive when the sound reflected from an obstacle is a broadband sound, while an obstacle shielding sound is suppressed and maintained by diffraction in the higher and lower frequency ranges, respectively [31,32,34,38]. However, the difference in the ease of perceiving obstacles depending on the shape of the sound source remains unexplored. Generally, the attenuation characteristics of point, line, and plane source sounds suggest that the interaural differences tend to be larger for point sources than for line or plane sources. Meanwhile, the interaural differences caused by the line and plane sound sources may be useful as a cue to perceive an obstacle, depending on the positional relationship between the listener, the obstacle, and the sound sources, as well as the way the listeners moves their heads. To clarify the actual situation of acoustic cues related to obstacle perception, further investigation is required because the present study did not confirm differences among the types of sound sources.

Moreover, the SPL of the experimental sound from the loudspeaker was adjusted to a level comfortable for the participants to listen to. While various studies on sound source localization have attempted to suppress artifacts by fixing the SPL value, this study employed no such control due to safety concerns as mentioned before. To clarify the effect of sound pressure level, it is necessary to set up an experimental system that ensures safety for visually impaired participants.

The number of participants was small compared to other psychological studies, despite being comparable to the number of participants in other related studies [31,33,38,44]. The reason for this was the limited number of blind people recruited for this study. Further studies are needed to explore the characteristics that may replicate the results of this study in a larger population of visually impaired people, as well as their hearing experiences and gait training.

## 7. Conclusions

First, obstacle distance localization by individuals who are blind was conducted in static and dynamic conditions. Consequently, the dynamic condition scores high accuracy in localized obstacle distance. From the result, introspection of participants, and a review of conventional studies, it was assumed that the interaural difference caused by head rotation during walking sensitizes the obstacle sense.

Subsequently, head rotation angle while the participants are walking and acoustic characteristics in relation to obstacle distance and head rotation angle were investigated. The head rotation angle during walking ranged from 2.78° to 11.11° (average: 6.55°, S.D.: 2.05°).

Regarding acoustic characteristics, acoustic coloration occurred and spectral interaural differences and interaural intensity differences were observed. Thereafter, the threshold of interaural differences in time (ITD), intensity (IID), and coloration (ICD) was investigated in blind participants. The results indicated that the corresponding head rotation angles of the ITD, IID, and ICD threshold were 23.5°, 9.17°, and 6.30°, respectively. Therefore, ICD was one of the most usable interaural difference cues facilitating the obstacle distance perception while walking.

These results provide a practical implication that visually impaired people can more accurately identify the distance to an obstacle by being aware not only of the sound changes that occur when walking close to an obstacle but also the interaural intensity differences (IIDs) and timbre change caused by interaural chromaticity differences (ICDs).

The remaining issue involves examining the relationship between acoustic changes due to head proximity and passive head movements and those due to active movements, and the importance of these factors in auditory obstacle localization by visually impaired individuals. Particularly, since multiple acoustic changes occur simultaneously in real environments, it is important to clarify which changes are mainly used by visually impaired people to perceive obstacles. Also, although this study was limited to blind persons, future work should also examine persons with severe low vision, who tend to rely on visual information to perceive obstacles using both auditory and visual perception, as well as the possibility of obstacle perception training using only auditory perception. We expect that clarification of these issues will lead to the development of auditory training methods and systems for improving the performance of obstacle sense in people with various visual disabilities.

## Data Availability

Data are contained within the article.

## References

[1] Ponchillia P.E., Ponchillia S.K.V. (1996). Foundations of Rehabilitation Teaching with Persons Who Are Blind or Visually Impaired.

[2] Ifukube T. (2017). Sound-Based Assistive Technology.

[3] Hakobyan L., Lumsden J., O’Sullivan D., Bartlett H. (2013). Mobile assistive technologies for the visually impaired. Surv. Ophthalmol..

[4] Hersh M.A., Johnson M.A. (2008). Assistive Technology for Visually Impaired and Blind People.

[5] Bhagotra S., Sharma A.K., Raina B. (2008). Psycho-social adjustments and rehabilitation of the blind. Soc. Med..

[6] Tuttle D.W., Tuttle N.R. (2004). Self-Esteem and Adjusting with Blindness: The Process of Responding to Life’s Demands.

[7] Miura T., Yabu K.i. (2023). Narrative review of assistive technologies and sensory substitution in people with visual and hearing impairment. Psychologia.

[8] LaGrow S., Wiener W., LaDuke R. (1990). Independent travel for developmentally disabled persons: A comprehensive model of instruction. Res. Dev. Disabil..

[9] LaGrow S.J., Weessies M.J. (1994). Orientation and Mobility: Techniques for Independence.

[10] Hill E.W., Ponder P. (1976). Orientation and Mobility Techniques: A Guide for the Practitioner.

[11] Long R.G., Hill E. (1997). Establishing and maintaining orientation for mobility. Found. Orientat. Mobil..

[12] Wiener W.R., Welsh R.L., Blasch B.B. (2010). Foundations of Orientation and Mobility, Volume 1.

[13] Ambrose G.V. (2000). Sighted children’s knowledge of environmental concepts and ability to orient in an unfamiliar residential environment. J. Vis. Impair. Blind..

[14] Huebner K., Merk-Adam B., Stryker D., Wolffe K. (2004). The National Agenda for the Education of Children and Youths with Visual Impairments, Including Those with Multiple Disabilities, Revised.

[15] Leong S. (1996). Preschool orientation and mobility: A review of the literature. J. Vis. Impair. Blind..

[16] Pogrund R., Rosen S. (1989). The preschool blind child can be a cane user. J. Vis. Impair. Blind..

[17] Silberman R.K. (2000). Children and Youths with Visual Impairments and Other Exceptionalities. Found. Educ. Hist. Theory Teach. Child. Youths Vis. Impair..

[18] Koenig A.J., Holbrook M.C. (2000). Foundations of Education, Volume II: Instructional Strategies for Teaching Children and Youths with Visual Impairments.

[19] Engel R.J., Welsh R.L., Lewis L.J. (2000). Improving the well-being of vision-impaired older adults through orientation and mobility training and rehabilitation: An evaluation. RE View.

[20] Long R., Boyette L., Griffin-Shirley N. (1996). Older persons and community travel: The effect of visual impairment. J. Vis. Impair. Blind..

[21] Hensley M. (1987). Rehabilitation in daily living skills: Effects on anxiety and self-worth in elderly blind persons. J. Vis. Impair. Blind..

[22] Kuyk T., Elliott J.L., Wesley J., Scilley K., McIntosh E., Mitchell S., Owsley C. (2004). Mobility function in older veterans improves after blind rehabilitation. J. Rehabil. Res. Dev..

[23] Diderot D. (1749). Thoughts on the Interpretation of Nature and Other Philosophical Works.

[24] Worchel P., Dallenbach K.M. (1947). “Facial Vision:” Perception of Obstacles by the Deaf-Blind. Am. J. Psychol..

[25] Supa M., Cotzin M., Dallenbach K.M. (1944). “Facial Vision:” the perception of obstacles by the blind. Am. J. Psychol..

[26] Kellogg W.N. (1962). Sonar System of the Blind: New research measures their accuracy in detecting the texture, size, and distance of objects “by ear.”. Science.

[27] Rice C.E. (1967). Human Echo Perception: Behavioral measurements are being made of human ability to detect objects by use of echoes. Science.

[28] Seki Y. (1998). Acoustical design of city for the visually handicapped. J. Acoust. Soc. Jpn..

[29] Ammons C.H., Worchel P., Dallenbach K.M. (1953). “Facial Vision”: The perception of obstacles out of doors by blindfolded and blindfolded-deafened subjects. Am. J. Psychol..

[30] Cotzin M., Dallenbach K.M. (1950). “Facial vision:” The role of pitch and loudness in the perception of obstacles by the blind. Am. J. Psychol..

[31] Seki Y., Ifukube T., Tanaka Y. (1994). Relation between the reflected sound localization and the obstacle sense of the blind. J. Acoust. Soc. Jpn..

[32] Seki Y., Ito K. (2003). Coloration perception depending on sound direction. IEEE Trans. Speech Audio Process..

[33] Miura T., Muraoka T., Ifukube T. (2010). Comparison of obstacle sense ability between the blind and the sighted: A basic psychophysical study for designs of acoustic assistive devices. Acoust. Sci. Technol..

[34] Miura T., Ifukube T., Furukawa S. Contribution of acoustical characteristics to auditory perception of silent object. Proceedings of the Systems, Man, and Cybernetics (SMC), 2011 IEEE International Conference on IEEE.

[35] Miura T., Okochi N., Suzuki J., Ifukube T. (2023). Binaural Listening with Head Rotation Helps Persons with Blindness Perceive Narrow Obstacles. Int. J. Environ. Res. Public Health.

[36] Wright H. (1963). Principles of auditory training for travel. Proc. Int. Congr. Technol. Blind..

[37] Welch J. (1964). A psychoacoustic study of factors affecting human echolocation. Res. Bull. Am. Found. Blind.

[38] Seki Y., Ifukube T., Tanaka Y. (1994). The influence of sound insulation effect on the obstacle sense of the blind. J. Acoust. Soc. Jpn..

[39] Nakamura-Funaba H., Ueda M., Iwamiya S. (2006). Questionnaire survey of the use of sound to support the mobility of the visually impaired. J. Acoust. Soc. Jpn..

[40] Rosenblum L.D., Gordon M.S., Jarquin L. (2000). Echolocating distance by moving and stationary listeners. Ecol. Psychol..

[41] Thurlow W.R., Runge P.S. (1967). Effect of induced head movements on localization of direction of sounds. J. Acoust. Soc. Am..

[42] Thurlow W.R., Mangels J.W., Runge P.S. (1967). Head movements during sound localization. J. Acoust. Soc. Am..

[43] Kato M., Uematsu H., Kashino M., Hirahara T. (2003). The effect of head motion on the accuracy of sound localization. Acoust. Sci. Technol..

[44] Schenkman B.N., Jansson G. (1986). The detection and localization of objects by the blind with the aid of long-cane tapping sounds. Hum. Factors.

[45] Blauert J. (1969). Sound localization in the median plane. Acta Acust. United Acust..

[46] Iwaya Y., Suzuki Y., Kimura D. (2003). Effects of head movement on front-back error in sound localization. Acoust. Sci. Technol..

[47] Brungart D.S., Kordik A.J., Simpson B.D., McKinley R.L. (2003). Auditory localization in the horizontal plane with single and double hearing protection. Aviat. Space Environ. Med..

[48] Ohuchi M., Iwaya Y., Suzuki Y., Munekata T. (2006). A comparative study of sound localization acuity of congenital blind and sighted people. Acoust. Sci. Technol..

[49] DeCarlo L.T. (2003). An application of a dynamic model of judgment to magnitude production. Percept. Psychophys..

[50] Green D.M., Luce R.D., Duncan J.E. (1977). Variability and sequential effects in magnitude production and estimation of auditory intensity. Percept. Psychophys..

[51] Wobbrock J.O., Findlater L., Gergle D., Higgins J.J. The aligned rank transform for nonparametric factorial analyses using only anova procedures. Proceedings of the SIGCHI Conference on Human Factors in Computing Systems.

[52] Elkin L.A., Kay M., Higgins J.J., Wobbrock J.O. An aligned rank transform procedure for multifactor contrast tests. Proceedings of the 34th Annual ACM Symposium on User Interface Software and Technology.

[53] Lenth R.V. (2016). Least-Squares Means: The R Package lsmeans. J. Stat. Softw..

[54] Lenth R., Singmann H., Love J., Buerkner P., Herve M. (2018). Emmeans: Estimated marginal means, aka least-squares means. R Package Version.

[55] Cohen J. (1992). A power primer. Psychol. Bull..

[56] Pettorossi V., Brosch M., Panichi R., Botti F., Grassi S., Troiani D. (2005). Contribution of self-motion perception to acoustic target localization. Acta-Oto-Laryngol..

[57] Brungart D.S., Rabinowitz W.M. (1999). Auditory localization of nearby sources. Head-related transfer functions. J. Acoust. Soc. Am..

[58] MacDougall H.G., McGarvie L.A., Halmagyi G.M., Curthoys I.S., Weber K.P. (2013). The video Head Impulse Test (vHIT) detects vertical semicircular canal dysfunction. PLoS ONE.

[59] Aggarwal J.K., Cai Q. (1999). Human motion analysis: A review. Comput. Vis. Image Underst..

[60] Müller S., Massarani P. (2001). Transfer-function measurement with sweeps. J. Audio Eng. Soc..

[61] Suzuki Y., Asano F., Kim H.Y., Sone T. (1995). An optimum computer-generated pulse signal suitable for the measurement of very long impulse responses. J. Acoust. Soc. Am..

[62] (2006). ISO 18233:2006 Acoustics—Application of New Measurement Methods in Building and Room Acoustics. https://www.iso.org/standard/40408.html.

[63] Kates J.M. (1985). A central spectrum model for the perception of coloration in filtered Gaussian noise. J. Acoust. Soc. Am..

[64] Hartmann W.M., Constan Z.A. (2002). Interaural level differences and the level-meter model. J. Acoust. Soc. Am..

[65] Giannouli V. (2013). Visual symmetry perception. Encephalos.

[66] Cattaneo Z., Fantino M., Silvanto J., Tinti C., Pascual-Leone A., Vecchi T. (2010). Symmetry perception in the blind. Acta Psychol..

[67] Kouchi M., Mochimaru M. (2005). AIST Anthropometric Database. Natl. Inst. Adv. Ind. Sci. Technol. H16PRO.

[68] Moore B.C. (2012). An Introduction to the Psychology of Hearing.

[69] Klumpp R., Eady H. (1956). Some measurements of interaural time difference thresholds. J. Acoust. Soc. Am..

[70] Henning G.B. (1974). Lateralization and the binaural masking-level difference. J. Acoust. Soc. Am..

[71] Hafter E.R., Dye R.H., Neutzel J.M., Aronow H. (1977). Difference thresholds for interaural intensity. J. Acoust. Soc. Am..

[72] Yost W.A., Dye R.H. (1988). Discrimination of interaural differences of level as a function of frequency. J. Acoust. Soc. Am..

[73] Bassett I.G., Eastmond E.J. (1964). Echolocation: Measurement of pitch versus distance for sounds reflected from a flat surface. J. Acoust. Soc. Am..

[74] Tsumura T. (1984). New methods in psychoacoustical measurements. J. Acoust. Soc. Jpn..

[75] Taylor M., Creelman C.D. (1967). PEST: Efficient estimates on probability functions. J. Acoust. Soc. Am..

[76] Lewald J., Karnath H.O. (2001). Sound lateralization during passive whole-body rotation. Eur. J. Neurosci..

[77] Lewald J., Karnath H.O., Ehrenstein W.H. (1999). Neck-proprioceptive influence on auditory lateralization. Exp. Brain Res..

[78] Feierabend M., Karnath H.O., Lewald J. (2019). Auditory space perception in the blind: Horizontal sound localization in acoustically simple and complex situations. Perception.

[79] Makous J.C., Middlebrooks J.C. (1990). Two-dimensional sound localization by human listeners. J. Acoust. Soc. Am..

[80] Miura T., Ebihara Y., Sakajiri M., Ifukube T. Utilization of auditory perceptions of sounds and silent objects for orientation and mobility by visually-impaired people. Proceedings of the 2011 IEEE International Conference on Systems, Man, and Cybernetics.

[81] Miura T., Ifukube T., Furukawa S. (2011). Analysis of acoustic cues for the auditory perception of silent object. J. Acoust. Soc. Jpn..

